# Internal diseases encountered by dental students while treating dental patients during undergraduate training

**DOI:** 10.1186/s12909-018-1258-3

**Published:** 2018-06-22

**Authors:** Anja Humbert, Petra Schmage, Sigrid Harendza

**Affiliations:** 1 Poliklinik für Zahnerhaltung und Präventive Zahnheilkunde, Universitätsklinikum Hamburg-Eppendorf, Hamburg, Germany; 2 III. Medizinische Klinik, Universitätsklinikum Hamburg-Eppendorf, Martinistr. 52, 20246 Hamburg, Germany

**Keywords:** Case-based learning, Dental education, Internal medicine, Learning objectives

## Abstract

**Background:**

The number of internal diseases, disorders and syndromes (IMDDSs) encountered in dental practice is increasing. Dentists report to feel ill prepared by their undergraduate dental training for the management of IMDDSs. To increase the effectiveness of internal medicine training at dental school it might be necessary to focus on IMDDs encountered by dental students. The aim of our study was to identify IMDDSs dental students come across while treating dental patients during the clinical years of their undergraduate training.

**Methods:**

All dental patients treated between April and July 2015 by 116 dental students enrolled at the Medical Faculty of Hamburg University in the semesters 7 to 10 were anonymously analysed retrospectively with respect to age, gender, smoking habits, drinking habits, current or previous diseases, allergies, current medication, dental diagnoses, and dental treatment in the current semester. Identified IMDDSs were clustered and evaluated.

**Results:**

The 116 dental students treated 511 patients with 559 IMDDSs with a median of one IMDDS per patient. The IMDDSs encountered most frequently could be assigned to the subspecialties cardiology, endocrinology/diabetology, and rheumatology. Arterial hypertension occurred most frequently in dental patients, followed by diabetes mellitus type 2, and chronic bronchitis.

**Conclusions:**

We identified the spectrum of IMDDSs encountered by dental students in the clinical years of their undergraduate dental education. Further studies are needed to test the effects of learning internal medicine with an internal medicine course based on the IMDDSs encountered by dental students and including additional IMDDSs specific relevance for dentists.

## Background

Internal diseases, disorders and syndromes (IMDDSs) occur more frequently with the increasing life expectancy of the population. Accordingly, the number of IMDDSs encountered in patients seeking dental treatment also increases and a high number of dentists reported difficulties in their dental practices due to insufficient knowledge of IMDDSs [1]. Many participants of a Japanese survey felt that internal medicine should have been covered more extensively during their dental education [1]. Dental students from Brazil felt insufficiently prepared by their dental school for IMDDSs and medical emergencies they might encounter in patients during dental treatment [2]. A retrospective study of dental patients’ records in India revealed that cardiovascular diseases accounted for the most prevalent internal medicine conditions in dental patients followed by endocrine disorders [3]. When internists and dentists were asked in a qualitative study which internal medicine topics should be taught at dental school, both groups mentioned cardiovascular diseases most frequently [4].

Dentists in private practice in Brazil reported an increasing number of emergencies with respect to cardiovascular diseases [5] and complications of IMDDSs in general [6]. When internists and dentists were asked to rank the IMDDSs compiled from these two studies according to their importance for dentists, coagulopathies, endocarditis, and anaphylaxis accounted for the top three topics to be taught in internal medicine at dental school [4]. Even though these studies underscore the demand to focus teaching of internal medicine during dental education on diseases and topics relevant for dentists, internal medicine teaching at medical faculties still seems to give rather a broad overview of internal medicine than providing students with a dentistry-oriented focus on specific diseases. Furthermore, it is not known which IMDDSs dental students encounter while treating dental patients themselves during the clinical years of their dental curriculum. A need therefore exists to determine the IMDDSs dental students are exposed to.

To identify the IMDDSs of dental patients treated by dental students might add another piece to the puzzle of creating learning objectives for the teaching of internal medicine at dental school. Therefore, the aim of our study was to determine the prevalence of internal diseases in dental patients treated by dental students in their clinical years at the dental school of the Medical Faculty of Hamburg University.

## Methods

In the dental curriculum at the Medical Faculty of Hamburg University dental students participate in an integrated clinical course of preventive, preservative, and prosthetic dentistry. This course includes 12 h per week and is held during the 14 weeks of semester 7 to 10. Learning objectives include taking dental patients’ histories, making dental diagnoses, and preparing dental treatment concepts. Students discuss these concepts with their supervisors before they carry out the dental treatment. To pass the course, students need to have completed a certain number of specific dental tasks, e.g. restorative treatment with fillings, root canal treatment, periodontitis treatment, and different prosthetic interventions. In addition to this practical course, dental students need to pass practical exercises in Orthodontics and Radiology as well as in Clinical Chemistry, Pathology, and Microbiology. Lectures on different dental and medical topics (e.g. IMDDSs) take place during semester 7 to 10 as well.

We retrospectively analysed all patients treated by dental students (semester 7 to 10) during their integrated clinical course of preventive, preservative, and prosthetic dentistry in the summer semester 2015, lasting from April to July at the Medical Faculty of Hamburg University. Of the 122 dental students enrolled in the semesters 7 to 10 in the summer semester 2015 at the Medical Faculty of Hamburg University, 116 (95%) consented in the anonymized analysis of the patients treated by them during this semester. Patients were included in this study, when a dental student treated them and the following criteria were documented: complete medical and dental history and complete dental findings including orthopantomogram. Patient data were gathered from the software system CHARLY® (Solutio, Hamburg), which contains all information about the patients treated by the students. The following patient characteristic were extracted from CHARLY®: age, gender, smoking habits, drinking habits, current or previous diseases, allergies, current medication, dental diagnoses, and dental treatment in the current semester. The following sociodemographic data about the students were collected: age, gender, and current semester. All data were anonymized for further evaluation. The Ethics Committee of the Chamber of Physicians of Hamburg confirmed that this study was in accordance with the Declaration of Helsinki. Data were grouped according to the characteristics defined above and internal diseases were categorized based on the ICD-10 system [7]. Results were reported descriptively using percent distributions.

## Results

Of the 532 patients treated by the 116 dental students (59.1% female) during the summer semester 2015, 511 (96.0%) fulfilled all inclusion criteria. Among the 511 patients included in the evaluation, 55% were male, 39% were smokers, and 4% were addicted to alcohol. The dental diagnoses of these 511 documented most frequently were caries (*n* = 468), periodontitis (*n* = 320), and insufficient dentition (*n* = 311). The dental treatments performed by the 116 students during the summer semester 2015 most frequently were dental fillings (*n* = 1002), removable and fixed dentures (*n* = 704), periodontology treatment (*n* = 512), and tooth extractions (*n* = 192).

**Fig. 1:**
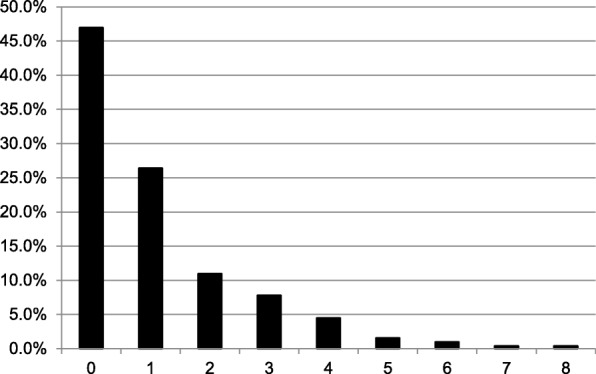
Percentage of the number of IMDDSs (0–8) per patient in the dental patients treated by dental students

**Fig. 2:**
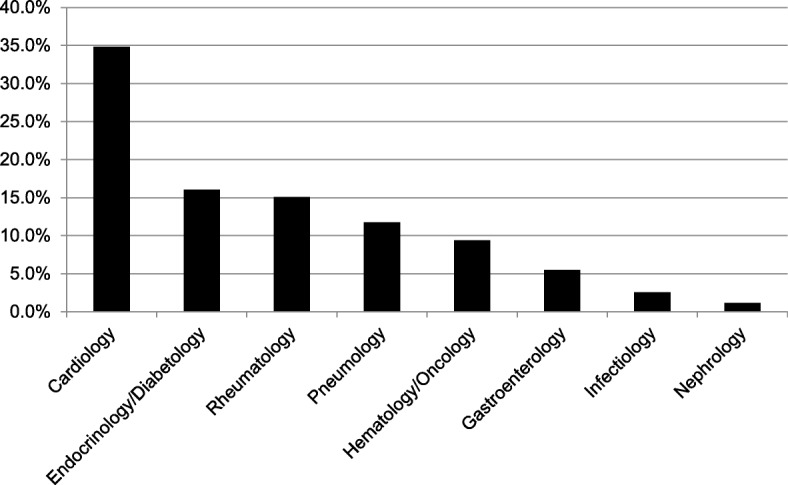
Percentage of IMDDSs per subspecialty

**Fig. 3:**
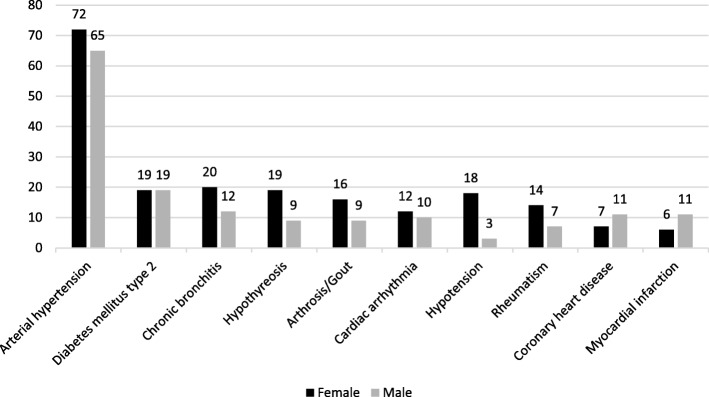
Frequency of IMDDSs in male and female patients

In total, 559 IMDDSs were documented in the 511 patients with a median of one IMDDS per dental patient treated by the students. Of the 511 patient, 240 patients (47.0%) had no IMDDS and 135 patients (26.4%) had only one IMDDS (Fig. 1). More than 30% of the IMDDSs could be assigned to the subspecialty Cardiology and approximately 15% each to the subspecialties Endocrinology/Diabetology and Rheumatology, respectively (Fig. 2). Arterial hypertension was the condition that occurred most frequently in female and male dental patients (*n* = 137), followed by diabetes mellitus type 2 (*n* = 38), chronic bronchitis (*n* = 32), hypothyroidism (*n* = 28), and arthrosis/gout (*n* = 25) (Fig. 3).

## Discussion

The diseases encountered most frequently by our dental students during the clinical years of their undergraduate curriculum are very similar to the most prevalent reported internal diseases of dental patients in the US, namely cardiovascular diseases, diabetes, hypertension, chronic bronchitis, and arthritis [8]. In a study from Thailand, cardiovascular diseases with the major component of arterial hypertension had the highest incidence in dental patients, followed by bone or joint disorders, and diabetes mellitus [9]. A study from India investigating the records of dental patients retrospectively for 1 year also discovered cardiovascular diseases to be the most prevalent conditions, which medically compromised dental patients, followed by endocrine disorders [3]. Chest pain associated with angina pectoris, diabetic events, and attacks of chronic bronchitis were the three most commonly experienced medical emergency events encountered in general dental practice in Great Britain [10]. Furthermore, the increase of certain internal diseases, e.g. diabetes mellitus, will cause an increasing number of dental diseases, e.g. periodontal disease, which will need treatment and require knowledge about these IMDDSs [11]. Therefore, we suggest that frequently occurring IMDDSs, especially when dental students already encounter them in their patients during their undergraduate clinical studies, should represent the centre of internal medicine teaching at dental school. Furthermore, it has been shown that structured reflection increased medical students’ situational interest in solving patient cases [12]. This might also motivate dental students to learn internal medicine and their experience with IMDDSs in their dental patients could help them to contextualise the theoretical knowledge as it has been shown for undergraduate medical students [13]. However, while learning based on self-encountered patient cases might increase the interest in internal medicine, it might not cover all IMDDSs [4] or medical emergencies [5] found to be relevant for dental practice.

In our study, the median of IMDDSs was one, but 136 patients suffered from two or more IMDDSs. An increasing number of internal diseases with the aging population in the twenty-first century was already anticipated in a study from the US in the 1980s [14] and holds true for many countries. For this reason, dental treatment also becomes more challenging in multimorbide patients and increasingly requires integrated clinical reasoning skills, which are often not taught during dental education [15]. Since knowledge seems to be organised in students’ minds in the same way it has been taught [16] learning internal disease connected with their occurrence in dental patients encountered in undergraduate clinical practice might be a favourable option because the need for authenticity in the learning process has been described to be a strategic requirement to teach clinical reasoning [17]. Inductive teaching and learning methods, which have been discovered to help dental students to develop clinical reasoning skills [18], might be a good strategy to memorize knowledge about internal diseases sustainably. Dental students preferred learning with cases in a problem-based way compared with lecture-based education [19]. Additionally, their knowledge of a chosen topic was higher than their knowledge of other topics [19]. To study internal diseases based on real patient cases encountered by dental students during the treatment of their dental patients might be a useful starting point to study specific IMDDSs relevant for future dentists. The IMDDSs could serve as tracer conditions for teaching and assessment [20, 21]. A similar learning approach of patient-based discussion ‘rounds’ has been suggested to teach biomedical science in dental education [22]. However, not all dental students will encounter the same IMDDSs when treating dental patients. For instance, dentals students in our school are not allowed to treat dental patients with certain diseases, e.g. hepatitis B. Yet knowledge about such IMDDSs still has to be acquired by dental students because of its relevance for their future practice. Therefore, other sources to define IMDDSs of relevance for dental practice need to be considered additionally when designing internal medicine teaching for dental students [4]. The IMDDSs encountered by the dental students while treating dental patients plus the other relevant IMDDSs could severe a starting points for case-based internal medicine teaching at dental school.

An increasing trend in the design of dental curricula was observed towards the creation of interdisciplinary curricula that are organized around themes [23] and towards integrated courses [24], which might be helpful to increase dental students’ understanding about the connection of certain dental and internal diseases. The idea to teach dental and internal medicine topics in an integrated course is not new. A course in internal medicine as part of the dental curriculum has been reported as early as 1935 [25]. Teaching IMDDSs in conjunction with dental topics has also been reported from the late 1960s to the 1980s from different countries [26–28]. An integrated course of internal medicine based on IMDDSs encountered by dental students during their clinical practice serving as tracer conditions [20] and based on IMDDSs relevant for dental practice in general, which students might not experience during their dental training [4], might be a worthwhile curricular change. Using IMDDSs as starting points for learning and discussing IMDDSs could improve dental students’ situational interest and motivation to learn internal medicine [12].

A weakness of our study is that it was performed at a single dental school, hence the interpretation cannot be generalized. However, the data were collected form dental students of different semesters, which is useful for the interpretation of the findings and increases the relevance of certain tracer conditions. Another weakness of our study is that we cannot be absolutely certain that all IMDDSs the patients were suffering from were documented in the computer system. A strength of our study is that it can be repeated easily at other dental schools, providing an approach to design an internal medicine course for dental education for an individual dental school’s needs. Another strength is that the results of our study provide information on the occurrence of IMDDSs during undergraduate dental education, a finding that can be used as a starting point for curricular design. Furthermore, several of the IMDDSs encountered frequently by our dental students play a role in adapting dental treatment to the specific needs of the patients [29], which could severe as a starting point for an interdisciplinary course design for internal medicine in dental education.

## Conclusions

We identified the spectrum of IMDDSs encountered by dental students during dental patients’ treatment in their clinical years of undergraduate dental education at the Medical Faculty of Hamburg University. These IMDDSs encountered by the dental students could serve as tracer conditions for an integrated case-based course of internal medicine for dental students to enhance their situational interest and motivation to study internal medicine. Other IMDDSs relevant for dentists according to the literature which might not have been encountered by the students themselves need to complete the course design. Eventually, such an internal medicine course design for dental students might lead to better knowledge about IMDDSs relevant for dental practice. Further studies are needed to test the effects of learning internal medicine with an internal medicine course based on IMDDSs relevant for dentists.

## Abbreviations

ICD: International Statistical Classification of Diseases and Related Health Problems
IMDDS: Internal diseases, disorders and syndromes
US: United States of America
